# Chronic Illness in Pediatric Critical Care

**DOI:** 10.3389/fped.2021.686206

**Published:** 2021-05-14

**Authors:** Sinead Murphy Salem, Robert J. Graham

**Affiliations:** Department of Anesthesiology, Boston Children's Hospital, Critical Care and Pain Medicine and Harvard Medical School, Boston, MA, United States

**Keywords:** chronic disease, critical illness, pediatric intensive care, chronic critical care illness, medical complexity, children special health care needs, technology dependence

## Abstract

Children and Youth with Special Healthcare Needs (CYSHCN), children with medical complexity (CMC), and children with chronic, critical illness (CCI) represent pediatric populations with varying degrees of medical dependance and vulnerability. These populations are heterogeneous in underlying conditions, congenital and acquired, as well as intensity of baseline medical needs. In times of intercurrent illness or perioperative management, these patients often require acute care services in the pediatric intensive care (PICU) setting. This review describes epidemiologic trends in chronic illness in the PICU setting, differentiates these populations from those without significant baseline medical requirements, reviews models of care designed to address the intersection of acute and chronic illness, and posits considerations for future roles of PICU providers to optimize the care and outcomes of these children and their families.

## Aims

- Define the subset of PICU patients with significant chronic illness and review their contribution to PICU epidemiology.
- List reasons for admission for this population.
- Consider care requirements and challenges unique to this population.
- Highlight innovations in care and healthcare systems.
- Discuss future direction of chronic care and PICU overlap.

## Introduction

Pediatric critical care medicine is traditionally considered a field of acute stabilization and management of quickly changing physiology. Though this description is certainly true, it is also a field that intersects with complex, chronic pediatric illness. The spectrum of chronic disease in children spans all ages, originating from congenital and acquired conditions, with a range of static, slowly resolving, and progressive declining trajectories. Such heterogeneity presents challenges for categorization, prediction modeling, research, bedside care, and support of families. This review article describes the spectrum of chronic illness, with examples, seen in the PICU, reasons for admission to the PICU for patients with chronic illness, perspectives on unique challenges to the PICU management, and innovative approaches of care for these populations.

## Defining Pediatric Chronic Illness

Numerous classification schemes have been developed over the years in an attempt to organize conversations and management of pediatric chronic illness and disability. The challenge of this endeavor lies in the diverse etiologies, single or multiorgan system involvement, prognoses, sequelae, and progression of various conditions in each individual patient. The current paradigm utilizes the following groupings as a nested model to relay the spectrum: children and youth with special healthcare needs (CYSHCN), children with medical complexity (CMC), and children with chronic critical illness (CCI). Though these categories include countless specific diagnoses, they often share common patterns of sequelae, healthcare support needs, or trajectories. Patients often move along this defined chronic disease spectrum over the course of a lifetime with slow improvements, disease progression, intercurrent illness, treatment responses, or technological advances.

### Children and Youth With Special Healthcare Needs

This broadest categorization encompasses children having, or at risk for, physical, developmental, emotional, or behavioral conditions requiring healthcare support beyond that of the general population (1, 2). This designation was developed in the 1990s to quantify the population of children with chronic healthcare needs and prioritize research surrounding this population (1). At that time, it was estimated that CYSHCN represented about 15% of the US pediatric population and accounted for 80% of all pediatric healthcare expenditures (2–4). This designation covers a very broad portion of the pediatric population, many of whom never require PICU care. CYSHCN may require intensive care hospitalizations at the time of an acute diagnosis that results in a chronic condition (e.g., presentation with malignancy or traumatic injuries) or at distinct points in chronic care management (e.g., an episode of diabetic ketoacidosis in a patient with known type 1 diabetes or status asthmaticus exacerbation).

### Children With Medical Complexity

Within the group of CYSHCN is this subgroup who have one or more chronic conditions associated with medical fragility, functional limitations, substantial outpatient service needs, and increased healthcare utilization (5, 6). This designation was highlighted in the early 2010's as the population of children with significant medial complexity, technology dependance, and multidisciplinary care needs expanded. Advances in perinatal care, resuscitative care, medications, technology supports, and changing attitudes toward decision-making over decades (7–9) contributed to the emergence of this population, and necessitated clear distinction from the larger population of children with chronic illness. It has been estimated that CMC represent one percent of the pediatric population in the United States, 10% of pediatric hospital admissions, 25% of hospital days, and 40% of hospital charges (4, 5). Examples of PICU admissions for CMC may include times of intercurrent illness (e.g., a children with static encephalopathy and gastrostomy tube dependance admitted with acute on chronic respiratory failure) or planned postsurgical care (e.g., after spinal surgery for a child with underlying neuromuscular disorder).

## Children With Chronic Critical Illness

In recent years, as children have survived intensive care with significant, persistent illness (10), this more narrow population has been defined by a history of a prolonged PICU stay, ongoing acute care needs, and dependance on technology, or persistent multiorgan dysfunction (11). The concept of chronic critical illness grew out of the adult literature (12, 13), but unlike adult patients with chronic critical illness who have 80% mortality within 1 year of hospital discharge (14), the majority of CCI survive multiple PICU admissions (15) with an estimate 5 years survival of 94% in pediatric long term ventilated patients (16). In many institutions, CCI require a PICU for all hospitalizations given fragile physiologic equilibrium and more intensive bedside care requirements. A CCI may require PICU hospitalization for routine care management (e.g., a child with chromosomal deletion, complex epilepsy, tracheostomy, and ventilator dependance admitted for antiepileptic titration) or seemingly mild illness in a patient with a tenuous baseline physiologic equilibrium (e.g., pneumonia in a former premature toddler with chronic lung disease, tracheostomy, and ventilator dependance).

- **Children and youth with special healthcare needs** have or are at risk for chronic physical, developmental, emotional, or behavioral conditions requiring healthcare support beyond that of the general population.
- **Children with medical complexity** have one or more chronic conditions associated with medical fragility, functional limitations, substantial service needs, and increased healthcare utilization.
- **Children with chronic critical illness** have history of a prolonged PICU stay, ongoing acute care needs, and dependance on technology or persistent multiorgan dysfunction.

## Demographics and Trends in the PICU

Within hospitals, and specifically PICUs, children with chronic illness represent an increasing proportion of the population and majority of healthcare utilization (17–20). It has been estimated that CYSHCN comprise more than 70% of PICU admissions (21) and are at three times greater risk for acute illness requiring PICU admission representing nearly half of all unscheduled admissions (17). CMC specifically accounted for nearly 50% of unscheduled PICU admissions (21). When considering both scheduled and unplanned PICU admissions, CMC represent the vast majority of all PICU days (78.8%), therapies including mechanical ventilation days (86.1%), and PICU costs (80.5%) (22). Patients without chronic illness received <10% of all PICU therapies (22). CYSHCN are at higher risk for prolonged length of stay (23), both early and late readmission (24, 25), medical errors (26), as well as in hospital mortality (27), compared to the general pediatric population. CCI, those with the most severe of chronic illness, have not been reported on with sufficient data, but it would be reasonable to speculate that given the degree of support required at baseline, these children alone represent a high degree of PICU needs.

## Pediatric Chronic Illness in the PICU

Indications for PICU admission among CYSHCN fall into, roughly, three categories: health maintenance for chronic conditions, perioperative management, and acute illness intervention. Each of these indications presents unique challenges and opportunities for improvement in healthcare delivery.

For patients with technology dependence or a constellation of intense home care regimens (e.g., oral suction needs, tracheostomy care, enteral and parenteral nutrition, and more extreme supports such as home inotrope infusions and ventricular assist devices), scheduled healthcare maintenance can often involve hospitalization and PICU stays. PICU level care is often required based upon hospital resource consolidation, risk for patient decompensation, and hospital staffing ratios. These scheduled care admissions (e.g., dental cleaning or screening bronchoscopy for tracheostomy/tracheal assessments) may necessitate anesthesia, pre-procedural optimization, overnight respiratory support titration, general uncertainty of response to procedural stress, and, occasionally, caregiver respite. A hospitalization, though necessary, poses risk to this vulnerable population during times of transportation, break from home routines for medications and medical cares, medication reconciliations (26, 28, 29), and rotating providers (30) not yet acquainted with the patient. These hospitalizations, though brief, can expose gaps in fragile home care networks with risk for disruption in home health staffing, medication and supply deliveries, or patient and family routines. Any of these disruptions can result in prolonged hospitalization for the patient (31).

Perioperative PICU admissions can be both elective and acute. CYSHCN are at increased risk for requiring acute surgical interventions related to congenital malformations, implanted surgical hardware (e.g., ventricular shunts, central venous access, enterostomy tubes), or complications and needs stemming from prior surgeries. Elective procedures may be scheduled to address sequelae of disease such as muscle contractures, scoliosis, or malnutrition. One tertiary children's hospital reported that CMC comprise 14% of operative cases (32) while another showed perioperative PICU admissions represent approximately half of all admissions for CMC with more than three quarters planned (22, 33). These stays tend to be prolonged compared to the general pediatric population with need for PICU monitoring and interventions. CMC are at higher risk compared to the general population for postoperative complications such as slow return of bowel function, hemodynamic instability, and respiratory insufficiency. In response to the perioperative needs of CYSHCN novel programs and guidelines in pre-operative evaluation, coordination of care, post-operative planning, and expectation management have been created (34).

Finally, acute illness is a major driver of PICU admission among CYSHCN and may include initial presentation with new illness, acute on chronic exacerbation of a chronic illness, or progression of underlying disease pathology. These conditions can be similar to the general PICU populations or very specific to the CYSHCN population. CYSHCN can be at risk for more severe presentation with acute illness given vulnerability due to chronic disease, delay in diagnosis, or impaired baseline organ function. These patients do not always follow expected disease progression or improvement models and experience longer PICU and hospital length of stays compared to the general pediatric population (23, 31).

## Population Specific PICU Considerations

Regardless of the indication for PICU admission, CYSHCN present population specific needs and challenges, differentiating them from children without pre-existing conditions. Heterogeneity among chronic conditions, extensive medication requirements, altered anatomy or physiology, and developmental needs can make PICU hospitalization challenging. Increased hospital exposure and infectious disease history can increase risk for antimicrobial resistance. Baseline organ dysfunction can require alterations in treatment algorithms (e.g., the threshold for starting antibiotics may be lower in a patient with short bowel syndrome compared to another patient of the same age). Developmental disabilities or behavioral diagnoses can make communicating pain and other symptoms difficult. Poor nutrition can delay wound healing. Anatomic abnormalities such as micrognathia and contractures can make common PICU procedures (e.g., airway management and vascular access) difficult.

PICU hospitalizations for CYSHCN can be prolonged due to factors beyond the patient's medical needs (23). Health systems factors such as home care requirements, home nursing coordination, supply deliveries, transportation logistics, and home facility availability can delay discharge for these patients (31). Family constraints such as caregiver training, resource navigation, and sibling support needs can also prolong hospitalizations (31). Finally, provider discomfort with limited experience meeting specialized needs for CMC or overall prognostication can impede discharge (31, 35, 36).

With increased chronicity and accompanying medical complexity, CYSHCN have large multi-disciplinary teams that can change frequently over the course of a PICU stay, creating challenges in collaboration, continuity, handoffs, medication reconciliation, ownership for medical decision making, and forming consensus (37, 38). Longer PICU stays may result as treatment plans are formulated and chronic medication regimens are manipulated (39, 40).

Family participation can serve as a cornerstone for PICU care of CYSHCN. Parents or primary caregivers can provide invaluable patient specific details including health summaries, interpretation of the patient's exam, rare disease specific expertise. At the same time, hospitalization can impart multifactorial stress on family caregivers. Prolonged PICU hospitalizations impart increased physical, emotional, financial, and logistical burdens. CCI are often hospitalized in tertiary centers far from family homes and community supports. Competing family responsibilities and geographic constraints can also limit patient-family relationships (41).

Acute care providers should appreciate that, despite chronic illness, families are often not prepared for acute clinical declines. and even death (42). Though a patient may live for some time with significant medical needs, “falling from that plateau” and landing in a PICU may result in significant emotional distress (43). It is important to establish family partnerships during the PICU stay and revisit goals of care for these patients, ideally prior to and throughout the PICU admission. Multidisciplinary supports as well as understanding are essential to tailoring family-centered care (44).

At the far end of the CYSHCN spectrum, CCI are a growing population at large and in the PICU. As CCI outstrip home and community resources and resources such as long term stay facilities are limited, some children are spending months or years in PICUs (41). The traditional PICU care model, with frequent provider transitions and nurses assigned to care for multiple patients, may not be optimal for the CCI PICU population. PICU logistics often emphasize quick bed turnover and staff priority caring for acutely evolving patients, at odds with patients who typically have longer recovery times, slower day to day progress, and maintenance requirements that are often delayed during acute hospitalizations (35, 41). Treatments such as physical therapy, developmental enrichment, social interaction, and experimental treatments (e.g., chemotherapy for a cancer patient on maintenance treatment that is limited by protocol restrictions or pragmatic considerations) are often paused during PICU admissions. At the same time, frequent vital sign monitoring and lab work common in the PICU may be overzealous in the care of CCI with prolonged recovery and contribute to poor rest and medical expenses. The PICU environment and mentality have evolved to address acute and, to an extent, subacute issues but in considering CCI with prolonged stays other quality of life metrics such as **s**ocial integration, long term health, family and community integration should not be neglected (45).

Moral distress and ethical complexities in care for CCI is another important aspect of care within this PICU population. This population shows a wide spectrum of disability, perceived quality of life, understanding of chronic pain, developmental potential, and autonomy. Providers, families, and the child himself may grapple with the establishing a goal for health restoration or maintenance, which have different connotations than a “cure” or return to “normal.” Advancing technologies (e.g., VADs), therapies (e.g., gene replacement or transcription modifiers), and novel surgical approaches coupled with enthusiasm may outpace our ability to inform the child and family of long-term outcomes, potential unforeseen risks, or pragmatic considerations for homecare. Multidisciplinary care with hospital resources such as palliative care, child life therapists, ethicists, social workers, and experienced PICU providers is imperative for the care of patient as well as the bedside team.

Survival for the PICU population has improved to the point that outcomes can no longer simply focus on mortality but must also address morbidity (46), such considerations apply to the CYSHCN, CMC, and CCI populations as well. Recovery trajectory, functional status, and quality of life are all important metrics for evaluation and prognostication within this population (47). Recent studies have demonstrated the impact of critical illness and PICU admission on patients and families (48, 49). Ongoing research highlighted in this journal are currently evaluating tools for outcome evaluation in CYSHCN and disabilities.

Special considerations for CYSHN in the PICU

- Does this patient have anatomic (e.g., airway or access), physiologic (e.g., pulmonary hypertension), or baseline abnormalities/condition-specific needs (e.g., inborn errors of metabolism) that will impact PICU care?
- How can we support the family both at the bedside and afar during prolonged hospitalizations?
- Are there other members of the child's continuity team that should be engaged for acute management as well as projected needs and care coordination?
- How can I support normalization of this patient's routine as he recovers from critical illness or awaits discharge? How can the family help us with PICU care? Are there skills transferable from the PICU to homecare that need to be taught or reinforced?
- What factors may impact discharge needs and timing beyond the patient's health (e.g., home nursing, new equipment, mobility considerations, school, or therapy accommodations)?
- hat is the child's *pre-PICU baseline*? What will life after PICU look like for this patient? Have we established a new health status or and altered recovery trajectory?

## Innovations in PICU Care for CYSHCN

As PICU providers of all disciplines and the healthcare system as a whole confront the changing landscape of pediatric chronic illness, several novel adaptations have been proposed to optimize safe, efficient, timely, and quality care. These innovations range from novel programs within traditional PICU models to alternative, parallel systems for the care of CYSHCN.

Within the traditional PICU model, some programs have created subgroups within the practice to address the care of CYSHCN. Examples include select groups of providers who care for pathology specific cohorts of patients such as surgical, oncologic (50), or neurologic (51) subgroups. Identification of PICU subgroups allows providers to become more familiar with emerging therapies, trajectory and care patterns, as well as specific needs of CYSHCN. Alternatively, some programs tailor services to “long-stay” cohorts based on frequency of PICU admission, with CCI cared for by select providers for the duration of the hospitalization to optimize continuity.

Many PICUs identify intensivists as primary continuity providers for patients with prolonged admissions to ensure communication with families, specialists, and outpatient providers (35, 37). Other practices have a consultation team of providers who give recommendations for CMC patients in the PICU, general inpatient, and primary care clinic. Similar to specialty specific consultation services, the primary objective of these teams is to provide overarching continuity while also supporting the primary team in symptom management and communication.

The opportunity for continuity in medical providers for CYSHCN at times of transition in care setting, whether within the hospital, transfer to another facility, or discharge home is of utmost importance as these patients have increased risk for miscommunication or decompensation during care transitions. Rare models incorporate a bridge team of providers who care for patients during acute critical illness, convalescence, and outpatient follow up (52, 53). More commonly, programs that incorporate continuity into hospital discharge rely on providers outside of the field of intensive care medicine, such as subspecialty specific, palliative care, or complex care general pediatricians (54).

For the care of CCI, specifically those requiring significant respiratory support, intensive care takes place in the patient's home and trials of home-visit based care have been successful in providing care for these patients. This model of care delivery allows for limiting patient transportation and disruption of routine, evaluation of patients in their home environments, and support of families within communities outside of large medical centers. Expansion of telemedicine options as well as partnership with local primary care, regional hospitals, and emergency medical services in future models will, ideally, decrease the frequency of PICU admissions within this population.

Intensivists are also working outside the PICU to ensure health of CYSHCN in consult roles with primary care physicians, inpatient complex care services, rapid response teams, and medical transportation teams. Institutions around the country are setting up post-PICU follow up programs and clinics to better understand and address the needs of CYSHCN after PICU discharge.

## Future Directions for Advancing Care

Pediatric critical care medicine is a field that addresses the health needs of children in their most vulnerable states. More than simply acute management and rapid change, it is a field at a crossroads with chronic illness and long-term medical needs. The opportunities and role for critical care may be less defined by PICU location going forward, as providers optimize care for increasingly complex cohorts of children beyond the PICU. The heterogeneity of the CYSHCN designation as well as the heterogeneity of the PICU population make this a difficult task. Continued emphasis on the intersection of these populations will allow for evidence-based advancements in care.

One important area of ongoing research is in the study of long-term outcomes of CYSHCN in PICUs. Understanding what life after discharge will look like for these patients should act as a cornerstone of PICU care. With better understanding of the impact of critical illness on development, social integration, family health, and physical health we can work as a field to mitigate risk incurred.

Finally, ongoing conversations must look for fresh approaches to optimal models of healthcare delivery for children with chronic illnesses in the PICU. The traditional model may not always make sense for the care of these patients, and we must be ready to adapt to fit the needs of a growing, complex, heterogeneous population.

## Author Contributions

SMS and RJG mutually contributed to the conception and design, literature review, drafting of the article, critical revision, and final approval of the version to be published. All authors contributed to the article and approved the submitted version.

## Conflict of Interest

The authors declare that the research was conducted in the absence of any commercial or financial relationships that could be construed as a potential conflict of interest.
